# Environmental Factors Related to Multiple Sclerosis in Indian Population

**DOI:** 10.1371/journal.pone.0124064

**Published:** 2015-04-22

**Authors:** Chaithra Malli, Lekha Pandit, Anita D’Cunha, Sharik Mustafa

**Affiliations:** Center for Advanced Neurological Research, KS Hegde Medical Academy, Nitte University, Mangalore, India; University of Oxford, UNITED KINGDOM

## Abstract

**Background:**

Multiple sclerosis (MS) is less prevalent among Indians when compared to white populations. Genetic susceptibility remaining the same it is possible that environmental associations may have a role in determining disease prevalence.

**Aims:**

To determine whether childhood infections, vaccination status, past infection with *Helicobacter pylori* (*H*.*pylori*), diet, socioeconomic and educational status were associated with MS.

**Material and Methods:**

139 patients and 278 matched control subjects were selected. A validated environmental exposure questionnaire was administered. Estimation of serum *H*.*pylori* IgG antibody was done by ELISA. Patients and controls were genotyped for HLA-DRB1*15:01.

**Results:**

In our cohort a significant association was seen with measles (*p* <0.007), vegetarian diet (*p* < 0.001, higher educational status (*p* <0.0001) and urban living (*p* <0.0001). An inverse relationship was seen with *H*.*Pylori* infection and MS (*p* <0.001). Measles infection (OR 6.479, CI 1.21- 34.668, *p*< 0.029) and high educational status (OR 3.088, CI 1.212- 7.872, *p*< 0.018) were significant risk factors associated with MS. *H*.*pylori* infection was inversely related to MS (OR 0. 319, CI 0.144- 0.706, *p* <0.005).

**Conclusions:**

Environmental influences may be important in determining MS prevalence.

## Introduction

The prevalence of Multiple sclerosis (MS) in India is likely to be around 8/100,000 [1] and is much lower than prevalence in white patients of European and North American ancestry. Among Indians the genetic susceptibility for MS appears similar to whites [2–3]. The principal risk allele for MS may be HLA DRB1* 1501. The established single nucleotide polymorphisms (SNP) identified in association with MS in white population appear to be similar for Indians. Environment is likely to play a significant role in MS disease pathogenesis.

Migration studies in the past have shown that genetic influences may not be enough to explain the change in risk of MS when patients migrate from low to high prevalence regions and vice versa [4]. Epidemiological data supports the “hygiene hypothesis” which was originally proposed to explain the incidence of MS in relation to sanitation in Israel [5]. According to this hypothesis exposure to several infections in childhood bolsters immunity and protects against later onset of MS with no specific agent being directly responsible [6–7]. In recent times it has been found that some infections found particularly among people of lower economic status and associated with poor hygiene may have a protective role. Typical examples include helminth [8] and *H*. *pylori* infection [9] which may exert immuno- modulatory effects that protects against later life autoimmune diseases. These factors may be relevant for the increased prevalence of MS in higher socioeconomic classes and in industrialized nations [10–11].

Environmental factors associated with MS in the west such as Epstein Barr virus (EBV) infection, Vitamin D deficiency and smoking [12] may not be risk factors for disease in the tropics. In India, by the age of 4 years > 90% of children are seropositive for EBV and cytomegalovirus (CMV) infection [13]. Not surprisingly there was no association found between MS and remote infection with EBV in Indian patients [14]. Most Indian women who are at greater risk of MS [15] are non smokers. Vitamin D deficiency is significant in the normal population [16]. A cross sectional study of Vitamin D levels in MS among Indians showed a risk association but reverse causality could not be excluded [17].

In the present study we have looked at the childhood infection profile of patients with MS and particularly the role of *H*.*pylori* infection. We have additionally evaluated factors that can potentially influence infection in childhood namely vaccination profile, socioeconomic and educational status, area of living and diet.

## Methods

### Patient and control selection

One hundred and thirty nine (92 female and 47 males) consecutive patients who fulfilled McDonald criteria 2010 [18] and had completed the environmental questionnaire were included. Patients were compared with 278 age and gender matched controls (Table 1). All patients were selected consecutively from the Mangalore demyelinating disease registry [19] at the second author’s (P.L) center in southern India. Healthy controls were patients who visited the outpatient clinic with minor neurological complaints such as headache or back pain and volunteered to donate blood.

**Table 1:** Demographic and Clinical features .

Demographic factors	MS (n = 139)	Control (n = 278)
Age (Mean± SD)	36.56± 11.95	36.69± 10.70
Gender	92 F/ 47M	184F/ 94M
Duration of disease (Median)	4 (3- 22yrs)	NA
Type of MS	RR = 78 SP = 61	NA
Ancestry	South Indian (non tribal)	South Indian (non tribal)

RR = Relapsing Remitting, SP = Secondary progressive, NA = Not applicable

#### Environmental exposure questionnaire

A detailed questionnaire (S1 Text) was used for both patients and healthy control subjects Prior validation (face and linguistic validation) was done and test- retest reliability was assessed (Cronbach’s alpha).

History of child hood (≤ 18years) infections such as chicken pox, mumps, measles and tuberculosis (these are disorders for which colloquial terms exist in local languages), vaccinations (as per national immunization schedule) and diet in childhood (vegetarian diet which included milk products versus non vegetarian diet) were noted. Socioeconomic background (low, middle and high Income groups were classified based on Prasad’s socioeconomic status calculation for Indians [20]) was determined. All patients and controls had attended school and were literate. Individuals who had attended college/ completed a college degree were identified from among patients and controls as being highly educated as opposed to those who attended / completed high school.

### Estimation of serum *H*.*pylori* IgG levels

Serum anti *H*. *pylori* IgG antibodies were detected by using Vircell (Granada, Spain) ELISA kits as per manufacturer’s instructions. The antibody index was determined by dividing the optical density values of the samples by the optical density for cut-off control samples and then multiplying by 10. Antibody index was considered positive if it is >11, equivocal if between 9 and 11 and negative if < 9. All equivocal results were retested and if found to remain equivocal the sample was reported as negative for *H pylori* IgG.

### HLA DRB1 genotyping

HLA DR typing was performed by polymerase chain reaction (PCR) with sequence specific probes [21]. Alleles that were DRB1*15:01/ 15:02 positive by this low resolution typing technique were sequenced for accurately determining HLA DRB1*15:01 status [22].

### Statistical methods

Statistical analysis was done using SPSS 20.0 (IBM corporation, Armonk, NY). Test—retest reliability of the environmental questionnaire (S1 Text) was calculated by running Cronbach’s alpha in SPSS. The frequency of *H*.*pylori* antibody seropositivity was compared between patients and controls. All categorical variables were analyzed by chi square (χ^2^) test. Keeping in mind the matched case and control design of the study a conditional logistic regression analysis was done in order to determine the risk association of environmental factors with MS. A *p* values < 0.05 was deemed to be significant. Odds ratios and confidence intervals were calculated.

### Standard protocol approvals, registrations, and patient consent

This study was approved by the institutional ethics committee (Central ethical committee, Nitte University). A written Informed consent approved by the institutional ethics committee was used for obtaining consent from every patient and healthy volunteer before blood sampling.

## Results and Discussion

The consistency/ reliability of the questionnaire was adequate (Cronbach’s alpha = 0.83). Among childhood infections measles (p < 0.007) showed strong association with MS (Table 2). Though statistically insignificant there were more number of patients with chicken pox (p <0.08) and tuberculosis (<0.19) in the patient group. Majority of patients and controls were vaccinated as per established national guidelines of the time (p < 0.63). The frequency of *H pylori* seropositivity was significantly low in MS patients as compared to controls (22.5% vs. 46%; *p* < 0.001).

**Table 2:** Environmental profile in Multiple Sclerosis patients and healthy controls.

Environmental factors	MS (n = 139) n (%)	Controls (n = 278) n (%)	*p* value
Diet—*Vegetarian*	33/139 (23.7%)	16/278(5.8%)	**0.0001**
Chickenpox	75/133(56.4%)	113/242(46.7%)	0.08
Measles	18/133(13.5%)	12/242(5%)	**0.007**
Tuberculosis	2/133(1.5%)	1/242(0.5%)	0.19
Vaccination (unvaccinated)	6/139 (4.3%)	9/278(3.2%)	0.63
*H*. *Pylori* IgG (positive)	31/139 (22.5%)	64/139(46%)	**0.001**
Socio economic status (middle class)	109/139(78.4%)	195/278(70%)	0.08
Educational status (graduates)	56/139 (40.3%)	41/278 (14.7%)	**0.0001**
Area of living (urban)	49/139(35.3%)	48/278(17.3%)	**0.0001**

MS patients were better educated than controls (p <0.0001) and lived mainly in urban areas (p <0.0001). Frequency of unvaccinated patients was <5% in both patients and controls. The economic background of both groups did not vary significantly though the middle income group was seen more in patients than controls (p <0.08). Those with high income were negligible in both patients and controls and hence were removed from analysis. HLA DRB1*15:01 allele was associated with MS patients (p < 0.02). When patients were stratified for DRBI* 15:01 allele, there was no significant association with *H*.*pylori* IgG serological status (data not shown).

Risk of disease was significantly associated with measles infection in childhood (OR 6.479, CI 1.21–34.668, *p<* 0.029). Higher educational status emerged as another risk factor (OR 3.088, CI 1.212–7.872, *p<* 0.018). Diet, socioeconomic status and area of living were not significant risk factors for MS (Table 3) nor was HLA DRB1 *15:01 status. There was a strong inverse relationship between risk of MS and *H*.*pylori* infection in our study which persisted after adjusting for other risk variables (OR 0. 319, CI 0.144–0.706, *p* <0.005).

**Table 3:** Environmental factors and risk of Multiple Sclerosis.

Factor	*p* value	Odds ratio	95% CI	
			Lower	Higher
*H pylori* IgG seropositivity	0.005	0.319	0.144	0.706
Diet	0.071	2.796	0.916	8.533
Socioeconomic status	0.698	0.851	0.377	1.921
Education	0.018	3.088	1.212	7.872
Area of living	0.523	0.744	0.300	1.842
Measles	0.029	6.479	1.211	34.668
DRB1*15: 01	0.131	1.852	0.832	4.124

In India there is an increasing shift of people from lower to middle income group urbanization and improved sanitary conditions. Autoimmune disorders such as type I Diabetes [23], Asthma [24] and thyroiditis have seen an increase [25]. Two decades ago the prevalence of MS in India was determined to be ≤ 1/ 100,000 [26]. Recently a population based survey has shown a prevalence nearly 8 times more than previous estimates [1]. After discounting for improved medical facilities and increasing awareness of the disease, there may be a true increase in MS prevalence in India.

Availability of suitable environmental triggers may determine the prevalence of autoimmune disorders such as MS given that genetic susceptibility is the same in most parts of the world [27]. This is the first comprehensive study evaluating childhood environmental factors related to MS in the Indian setting. Our data gathering was mostly questionnaire based and hence recall bias cannot be ruled out. Our sample size was small. Despite these limitations in this study a significant association was seen with child hood measles infection. In addition a higher socio economic status, living in urban regions and a vegetarian diet was noted in MS patients but not healthy controls. A significant negative association was seen with *H*.*pylori* seropositive status in MS patients. The risk factors that emerged for MS in our cohort were measles infection in childhood and high educational status. Lower seroprevalence of *H*.*pylori* emerged as a strong protective factor after adjusting for other variables.

The association of measles is well known in MS among whites [28]. In MS a polyspecific response of B cells occurs against a variety of neurotopic viruses particularly measles (MRZ reaction) [29]. However its possible role in disease causation in MS is not very clear. There are also studies which found no association between childhood viral infections including measles and later life MS [30].

A possible association of measles with MS in nonwhite populations has been brought out in our study. This is in sharp contrast to the lack of association with EBV as a risk factor for MS among Indians [14]. The protective effect of *H pylori* infection seen in our study has been previously reported from Japan [9, 30] and Iran [31]. *H*.*pylori* occurs in the stomach in > 50% of entire human population and may be as high as 80–90% in tropical settings [32]. Infection occurs predominantly before 2 years of age before parietal cells start secreting gastric acids that hamper the survival of the organism [33]. Once acquired the bacterium persist for decades. Thus difference in frequency of *H*.*pylori* seropositivity gives an indirect measure of the infectious / sanitary environment in childhood. It is possible that immunomodulatory effect of chronic *H*.*pylori* infection is protective against MS. Significantly *H*.*pylori* serological status was not influenced by DRB1*15:01 status in our study. The strong association with urban living and high educational status seen in our study supports the protective role of *H*.*pylori* and the importance of the hygiene hypothesis in disease causation. The role of dietary factors may be important. MS patients in our cohort were significantly vegetarians from early childhood. While dietary deficiencies (especially Vitamin D) may be partly responsible, the effect of diet in modulating the gut micro biome in early life and in turn the innate immunity is a plausible alternative to be considered [34,35].

## Conclusions

The established environmental risk factors for MS in white populations may not be the same for nonwhites. Published data on African Americans [36], Hispanics [37] and Indians [17,19] support this notion. Larger studies from Indians and other ethnic populations with low to moderate MS prevalence are important to understand early childhood environmental influences that not only confer disease susceptibility but also offer protection. Knowledge of these factors may improve our understanding of the varied prevalence of MS worldwide.

## Supporting Information

S1 Text Questionnaire with Environmental factors. (PDF)
