# Supplementary material for: Environmental Factors Related to Multiple Sclerosis in Indian Population
Source: PLoS One. 2015 Apr 22;10(4):e0124064. doi: 10.1371/journal.pone.0124064 (PMC4406747; doi:10.1371/journal.pone.0124064)
Supplement: S1 Text — (PDF) [file pone.0124064.s001.pdf]

# Questionnaire with Environmental factors

## Case

### Clinical Data- Part 1 (Demographic)

Center Date of entry

Name Age Sex

Weight (Kg) Height (mts) BMI

Address Contact numbers

Religion Caste Language

Date of birth (d/m/yr) - Place of birth

Migration history – No

Yes - Migrated from To

Age at migration

Educational status: Illiterate Primary High school PUC Graduate

Monthly Income: Low (<Rs4000/m), Lower Middle ( 4000-15,000/m),

Upper Middle ( Rs 15,000- 55000/m) , high (> 55000/m)

Diet: Veg Non Veg

Area of living - Rural Urban Slum

**Clinical Data –Part 11 (Personal habits)**

**A. Smoking**

Has the patient ever smoked cigarettes?

No / Yes If yes, age of smoking onset: (yrs)

Number of cigarettes per week:

If the patient no longer smokes, age at stopping (yrs)

Has anyone in the patient's household smoked while living with him/her?

No / Yes If yes, how many people smoke?

How old was the patient when exposed to second-hand smoke

in the home? ( yrs )

How many cigarettes per day are/ were consumed (on average) in the patients household?

If no-one in the home smokes now, age of patient when smoking stopped:

| **B. Other habits** | Yes | No | duration |
|---|---|---|---|
| Betel nut - | | | |
| Tobacco chewing - | | | |
| Snuff - | | | |

**C. Concurrent illness -**

A. Diabetes No Yes Age of diagnosis (yrs)

B. Auto immune disease- (Tick against) Bronchial asthma, Allergy, Thyroiditis, Collagen vascular Disease, other (specify)

**D. Past History of infections** (Tick against)- Don't remember

Remember - Measles, Mumps, Chicken Pox, TB, other

**E. Vaccinations** –Don't remember

Remember - Measles, Mumps, Rubella, DPT,Polio, TB, Hepatitis

F.**Family history-**

a) MS-

b) Other Neurological disorder (Name of disease and relative affected)

| Mother | Father | Sibling | Other Relative | |
|---|---|---|---|---|
| | | | Maternal<br><br>Relation | Paternal<br><br>Relation |
| ☐ | ☐ | ☐ Sib#, -------------<br><br>☐ Sib#, ------------- | ☐ Uncle<br><br>☐ Aunt<br><br>☐ Grandmother<br><br>☐ Grandfather | ☐ Uncle<br><br>☐ Aunt<br><br>☐ Grandmother<br><br>☐ Grandfather |

## G. Sun Exposure Information

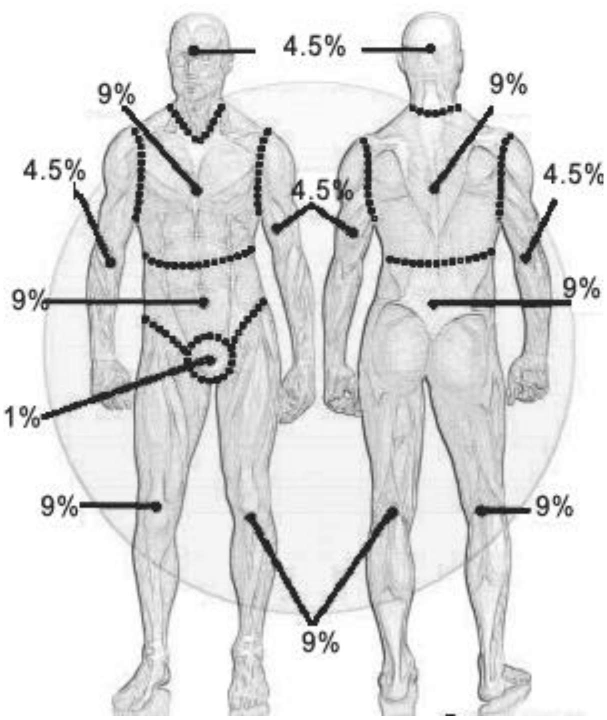


| Dress code | | Morning<br><br>(8.00 am to 1.00 pm) | Afternoon<br><br>(1.00 pm to 5.00 pm) | Evening<br><br>(5.00 pm to 6.00 pm) | Total Hours of Sun Exposure | Percentage of Sun Exposure |
|---|---|---|---|---|---|---|
| | Ages 6 to 12 | | | | | |
| | Ages 13 to 15 | | | | | |
| | Ages 16 to 18 | | | | | |
| | Ages 19 to 21 | | | | | |
| | 22 years and up | | | | | |
| | | | | | Mean= | Mean= |

## Healthy control

**Name** **Age/ Sex**

**Address** **Contact Number**

Weight (kg) Height (mts) BMI

Relation to patient - Spouse , Friend, Medical personnel, Patient volunteer, other

Primary diagnosis (for patient volunteer)

**Religion** **Caste** **Language**

**Educational Status**: Illiterate, Primary, High School, PUC, Graduate

**Area of living** - Rural Urban Slum

**Diet:** Veg Non veg

**Monthly Income :** Low (<Rs4000/m) , Lower Middle ( 4000-15,000/m),

Upper Middle ( Rs 15,000- 55000/m) , high (> 55000/m)

**History of** 1. Diabetes 2. Asthma 3. Allergies 4. Thyroiditis 5. Other Automimmune disease (specify)

**Past Infections** : Don't remember

Remember - Measles, Mumps, Chicken Pox, TB, other

**Past vaccinations** – Don't remember

Remember - Measles, Mumps, Rubella, DPT, Polio, TB, Hepatitis

**Smoking** – Has the control ever smoked cigarettes?

No / Yes If yes, age of smoking onset: (yrs)

Number of cigarettes per week:

If the control no longer smokes, age at stopping (yrs)

Has anyone in the controls household smoked while living with him/her?

No / Yes If yes, how many people smoke?

How old was the control when exposed to second-hand smoke

in the home? ( yrs )

How many cigarettes per day are/ were consumed (on average) in the controls household?

If no-one in the home smokes now, age of control when smoking stopped:

| **Other habits** | Yes | No | duration |
|---|---|---|---|
| Betel nut - | | | |
| Tobacco chewing - | | | |
| Snuff - | | | |

## Sun Exposure Information

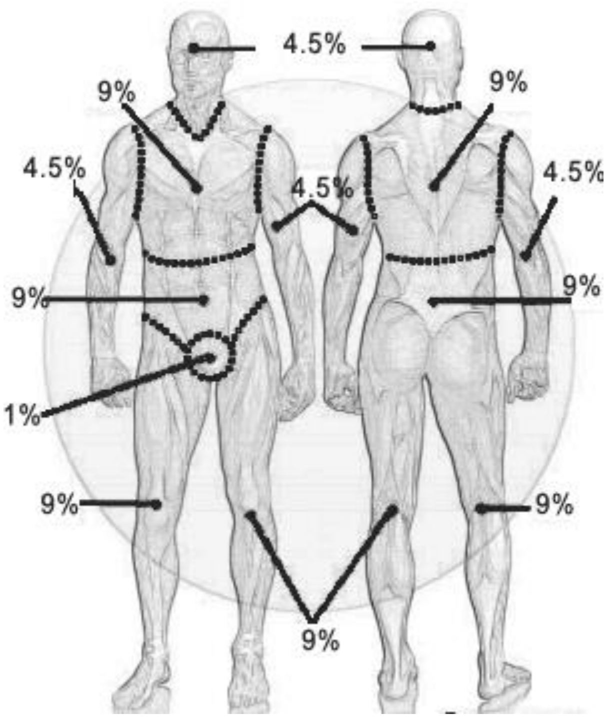


| Dress code | | Morning<br><br>(8.00 am to 1.00 pm) | Afternoon<br><br>(1.00 pm to 5.00 pm) | Evening<br><br>(5.00 pm to 6.00 pm) | Total Hours of Sun Exposure | Percentage of Sun Exposure |
|---|---|---|---|---|---|---|
| | Ages 6 to 12 | | | | | |
| | Ages 13 to 15 | | | | | |
| | Ages 16 to 18 | | | | | |
| | Ages 19 to 21 | | | | | |
| | 22 years and up | | | | | |
| | | | | | Mean= | Mean= |
